# A review on microbes mediated resource recovery and bioplastic (polyhydroxyalkanoates) production from wastewater

**DOI:** 10.1186/s12934-024-02430-0

**Published:** 2024-07-01

**Authors:** Vishal Ahuja, Pankaj Kumar Singh, Chandan Mahata, Jong-Min Jeon, Gopalakrishnan Kumar, Yung-Hun Yang, Shashi Kant Bhatia

**Affiliations:** 1 Department of Biotechnology, University Centre for Research & Development, Chandigarh University, Mohali, Punjab 140413 India; 2 Department of Agricultural and Biological Engineering, University of Illinois at Urbana- Champaign, 1304 W. Pennsylvania Avenue, Urbana, 61801 USA; 3 Green & Sustainable Materials R&D Department, Research Institute of Clean Manufacturing System, Korea Institute of Industrial Technology (KITECH), Chungnam, 331-825 Republic of Korea; 4 School of Civil and Environmental Engineering, Yonsei University, Seoul, 03722 Republic of Korea; 5 Institute of Chemistry, Bioscience and Environmental Engineering, Faculty of Science and Technology, University of Stavanger, Box 8600, Forus, Stavanger, 4036 Norway; 6 Department of Biological Engineering, College of Engineering, Konkuk University, Seoul, 05029 Republic of Korea; 7 Institute for Ubiquitous Information Technology and Application, Konkuk University, Seoul, 05029 Republic of Korea

**Keywords:** Bioplastic, Polyhydroxyalkanoates, Wastewater, Downstream processing

## Abstract

**Background:**

Plastic is widely utilized in packaging, frameworks, and as coverings material. Its overconsumption and slow degradation, pose threats to ecosystems due to its toxic effects. While polyhydroxyalkanoates (PHA) offer a sustainable alternative to petroleum-based plastics, their production costs present significant obstacles to global adoption. On the other side, a multitude of household and industrial activities generate substantial volumes of wastewater containing both organic and inorganic contaminants. This not only poses a threat to ecosystems but also presents opportunities to get benefits from the circular economy.

**Main body of abstract:**

Production of bioplastics may be improved by using the nutrients and minerals in wastewater as a feedstock for microbial fermentation. Strategies like feast-famine culture, mixed-consortia culture, and integrated processes have been developed for PHA production from highly polluted wastewater with high organic loads. Various process parameters like organic loading rate, organic content (volatile fatty acids), dissolved oxygen, operating pH, and temperature also have critical roles in PHA accumulation in microbial biomass. Research advances are also going on in downstream and recovery of PHA utilizing a combination of physical and chemical (halogenated solvents, surfactants, green solvents) methods. This review highlights recent developments in upcycling wastewater resources into PHA, encompassing various production strategies, downstream processing methodologies, and techno-economic analyses.

**Short conclusion:**

Organic carbon and nitrogen present in wastewater offer a promising, cost-effective source for producing bioplastic. Previous attempts have focused on enhancing productivity through optimizing culture systems and growth conditions. However, despite technological progress, significant challenges persist, such as low productivity, intricate downstream processing, scalability issues, and the properties of resulting PHA.

**Graphical abstract:**

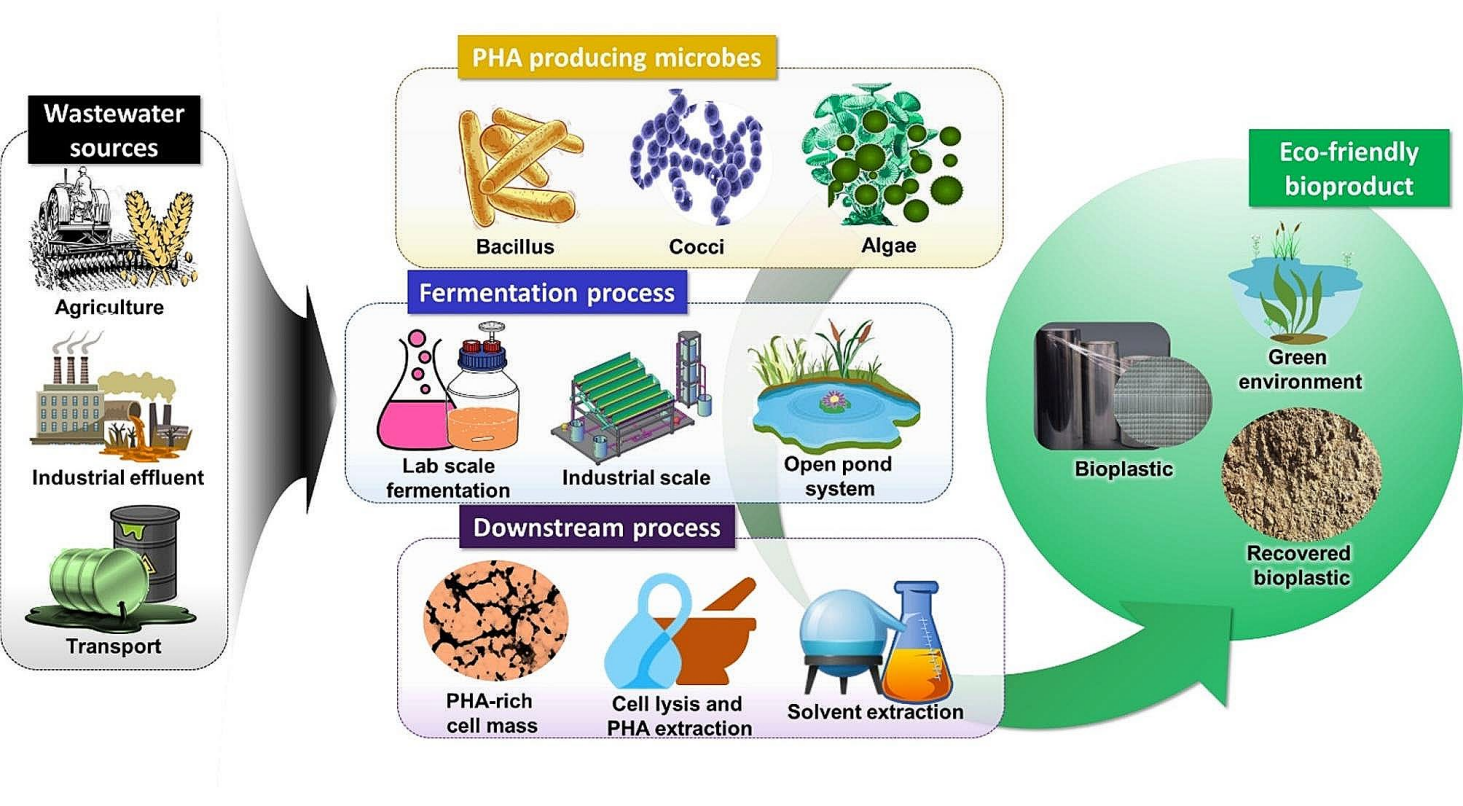

## Background

Plastic waste and environmental pollution pose significant global challenges, stemming from a dramatic increase in production and usage over recent decades [1]. Primary plastic production globally surged from 9,200 million metric tons (1950–2017) to a projected 34 billion metric tons by 2050 [2]. A critical concern lies in plastic’s enduring presence in landfills, water bodies, and ecosystems [3]. Of the total 7 billion tons of plastic waste generated, less than 10% undergoes recycling, while 14% is incinerated, leaving 76% to either accumulate in landfills or infiltrate natural environments [2]. Annually, approximately 14 million metric tons of plastic find their way into the ocean, resulting in an estimated 170 trillion plastic particles dispersed throughout the world’s oceans. Experts predict this figure to triple within the next two decades [4].

Plastic waste undergoes degradation through physical, chemical, and biological processes. Physical and mechanical forces work to reduce particle size, while energy transfer, like heat weakens structural integrity and functionality [5–7]. Chemical methods, including exposure to UV radiation, ozonolysis, or catalytic conversion, induce chemical changes that diminish mechanical strength, cause embrittlement, and release residues [5, 7]. The biological degradation (biodegradation) of plastics, considered a safer process occurs under the influence of microbial (bacteria, fungi, and algae) metabolism and enzymatic action. This phenomenon can be seen distinctly in various environments including landfills, oceans, and soil [8]. First microbes colonize on particles and secrete enzymes to depolymerize polymers and then use them as a carbon source. Microorganisms employed various enzymes including PETase, esterase, lacasses, cutinases, etc., for plastic degradation by acting on carbon back bone, side chains, and hetero atoms [9]. From the mangrove sediments, Auta et al. (2018) isolated *Bacillus* sp. strain 27 and *Rhodococcus* sp. strain 36, reporting 6.4% and 4.0% polypropylene (PP) degradation in 27 and 40 days, respectively [10]. Many other microbes have been reported to degrade various plastics including *Pseudomonas putida* IRN22, *Acinetobacter pittii* IRN19, *Micrococcus luteus* IRN20 [11] *Pseudomonas citronellolis* [12]. Besides microorganisms, *Tenebrio molitor* larvae have also been reported to depolymerise the polyvinyl chloride [13]. However, these processes are slower and result in the generation of plastic traces, including microplastics and nanoplastics, which can easily infiltrate marine and terrestrial environments, posing risks to both humans and animals [8, 14]. Ingestion or inhalation of microplastics may lead to oxidative stress, inflammatory reactions, and metabolic disorders [14, 15].

Various biobased materials, including starch, chitin, chitosan, and cellulose, have been explored for their potential in packaging applications [16–18]. Polyhydroxyalkanoates (PHA) emerge as a promising alternative to fossil-based plastics, offering comparable strength and environmental friendliness [19]. However, the cost-effectiveness of PHA production is predominantly influenced by feedstock expenses and product recovery, with nearly 40% of the cost attributed to feedstock and facing issues with low yield [20, 21]. In this context, waste resources such as wastewater from various sources including municipal, industrial, and agricultural practices present a viable and cost-effective alternative for bioplastic production, while also enabling resource reclamation [22–24]. According to United Nations reports from 2023 [25] a total of 320 billion m^3^ of wastewater is generated globally. More than 70% of freshwater is utilized for agricultural purposes, while industries consume 22% [26]. Only 11% of wastewater from domestic and industrial sources is earmarked for reuse, leaving over 42% of household wastewater partially treated. The wastewater generated alone accounts for approximately 1.57% of greenhouse gas (GHGs) emissions [25]. Wastewater from various sources contains high organic content, such as sugars and fatty acids, which can be harnessed by microorganisms for the production of PHA [27]. Numerous studies have explored PHA production using diverse substrates, including municipal wastewater [28], cheese whey by anaerobic mixed culture [29], olive oil mill wastewater by acidogens [30], secondary wastewater sludge, and waste sludge [31], hardwood sulfite spent liquor [32], paper industry effluent [33], raw sludge by methanotrophs [34], phototrophic mixed culture [35] and by anaerobes [36]. These studies highlight the versatility of wastewater as a substrate for PHA production, showcasing its potential to revolutionize the bioplastic industry by utilizing diverse waste streams.

Recovering PHA from fermentation broth poses another challenge in the production process. Traditionally, recovery methods involve either cell lysis or solvent-based extraction. The conventional approach favors chloroform (CHCl_3_) as a standard solvent [37]. However, alternative methods, such as antisolvent systems [38] using nonhalogenated solvents, have been explored to extract PHA. Concerns over environmental and user toxicity have steered the search for greener and non-toxic solvents. Advanced techniques have introduced biobased solvents like 3-hydroxybutyrate-*co*-3-hydroxyvalerate and methyltetrahydroxyfuran (2-MTHF) [39], as well as dimethyl carbonate, ethanol, ethyl acetate, ethyl lactate, and methanol [40], which have proven to be equally effective in extraction without toxic effects compared to the standard process. The primary considerations for downstream processing remain environmental impact and overall cost which can be achieved by use of greener solvent-based extraction, solvent recycling, and revalorization of residues. The exploration of bioplastics has garnered significant attention in both research and industrial sectors. Previous literature has examined various facets of bioplastics, including their environmental benefits, production from different waste sources such as food, vegetables, downstream processing, and stability in different environments. For instance, Lavagnolo et al. [41] delved into the stability of bioplastics in aquatic environments, while Gong et al. [42] and Ali et al. [43] summarized bioplastic production from fruit-vegetable residues and organic waste, respectively. Additionally, Bhat et al. [44] addressed greener approaches for bioplastic recovery. However, there is limited information available on utilizing wastewater as a feedstock. The current article comprehensively covers various aspects from feed conversion to bioplastic production, including techno-economic analysis and cost considerations.

## Microbial PHA synthesis pathways

**Fig. 1:**
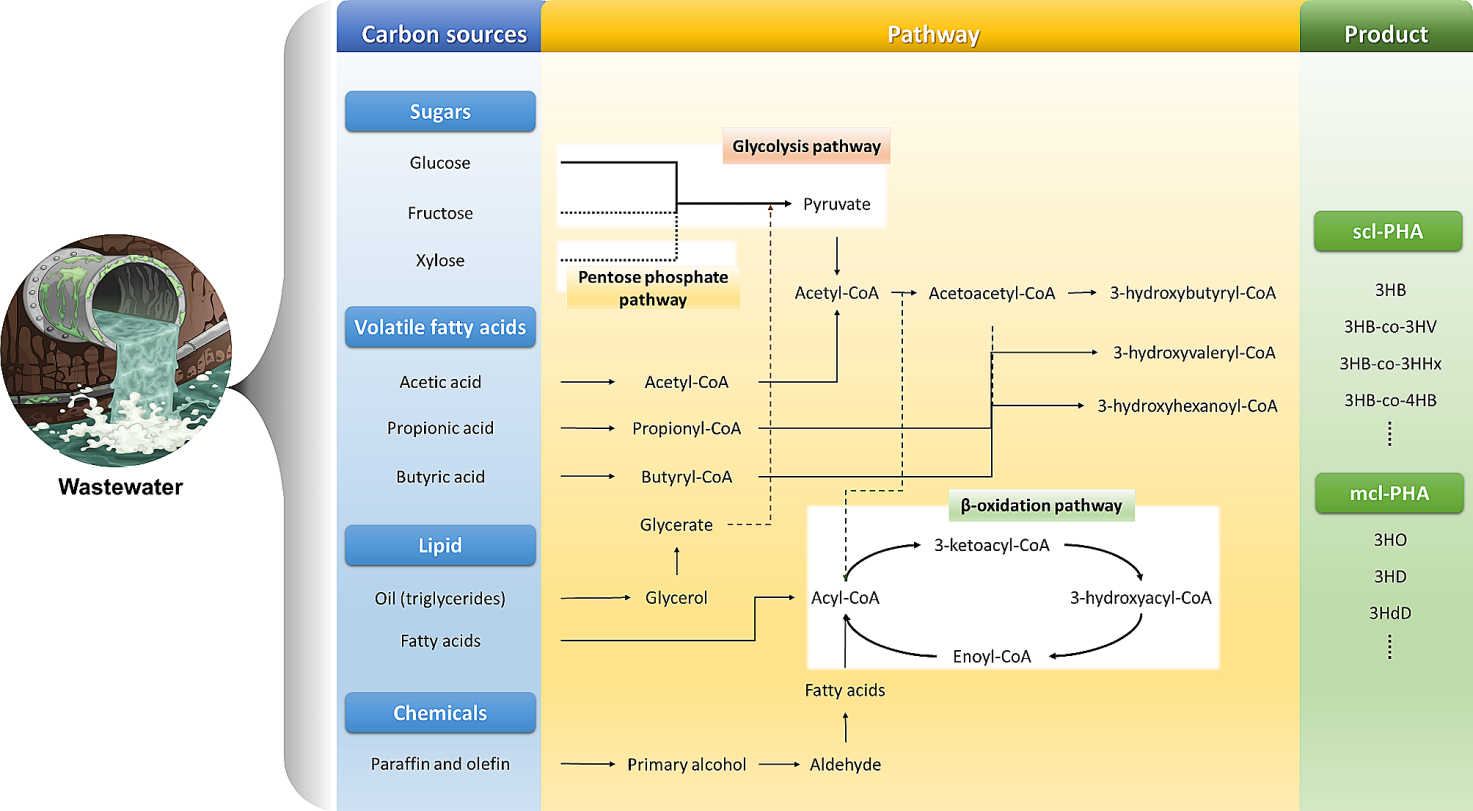
Different pathways for the production of bioplastic from pollutants in wastewater

The microbial synthesis of PHA stands as a remarkable example of nature’s innovation, offering a sustainable avenue for producing biodegradable bioplastics [45, 46]. Microorganisms produce PHA as intracellular carbon reserves in response to nutrient imbalances, particularly an excess of carbon and limited nitrogen or phosphorus, storing them as granules [18, 47]. The biosynthetic operon for PHA consists of a cluster of genes, including PHA synthase, *β*-ketothiolase, and NADPH-acetoacetyl-CoA reductase, organized in close proximity [48, 49]. Carbon sources are converted into acetyl-CoA, a precursor for PHA synthesis, through pathways such as glycolysis or beta-oxidation of fatty acids [50]. The synthesis of polyhydroxybutyrate (PHB) involves the condensation of two acetyl-CoA molecules to form acetoacetyl-CoA, catalyzed by the enzyme β-ketothiolase. Subsequently, acetoacetyl-CoA is reduced to (R)-3-hydroxybutyryl-CoA by (R)-specific acetoacetyl-CoA reductase. Finally, (R)-3-hydroxybutyryl-CoA is polymerized into PHB by the enzyme PHA synthase [51, 52]. This pathway utilizes the organic components available in wastewater, such as oil, volatile fatty acids (VFAs), glucose, fructose, xylose, and amino acids, transforming them into PHA and intermediates (Fig. 1).

The acidogenic effluent derived from dairy waste presents itself as a cost-effective, acid-rich wastewater stream suitable for producing PHA [53]. In a study by Pagliano et al. [54], the efficacy of dairy wastewater-derived effluent for PHB production in specific biobased industrial applications was demonstrated, achieving a significant PHB content of 0.31%, twelve times higher than the control group at the same time (0.025%). *Cupriavidus necator* DSM 13,513 was identified as the most efficient strain for accumulating PHB from the fatty acid-rich effluent of an anaerobic process fed with dairy wastewater. Other research endeavors have successfully produced PHB using effluents from waste sewage and food industry wastewater [55]. Furthermore, studies indicate the potential utilization of food industry wastewaters, such as confectionery wastewater (CWW) and rice parboiling water (RPW), as cost-effective substrate sources for PHB production [56].

Microorganisms possess the capability to utilize oils and produce medium-chain-length polyhydroxyalkanoates (*mcl*-PHA) [57]. (R)-3-hydroxyacyl-CoA, an intermediate of fatty acid metabolism, directs the conversion of fatty acids into PHA. By bypassing the fatty acid *β*-oxidation pathway, acetyl-CoA and de novo pathway intermediates are directed towards PHA biosynthesis. Transacylase, a critical enzyme in PHA biosynthesis, transfers the (R)-3-hydroxyacyl moiety from the respective acyl carrier protein (ACP) thioester to CoA [58, 59]. Notably, the genes encoding transacylase and enoyl-CoA hydratase are co-regulated but are not situated within the PHA synthase operon [60, 61]. *Bacillus thermoamylovorans* strain PHA005, isolated from palm oil mill wastewater effluent, demonstrates the ability to produce *mcl*-PHA at 50.77% of dry cell weight (DCW), which increases to 63.27% under optimal growth conditions, including a C/N ratio of 5:1 at 45 °C [62]. Mixed microbial cultures (MMCs) have been utilized for PHA production, yielding both short-chain-length (*scl*-PHA) and *mcl*-PHA using enzymatically pretreated palm oil wastewater as feedstock [63]. The success of PHA production from wastewater relies on the microorganisms’ capacity to accumulate intermediates and polymers. To enhance this process, enrichment techniques can be employed. MMCs enriched with a mixture of volatile fatty acids (VFA_mix_) have shown improved accumulation results, with the maximum reported accumulation capacity achieved using the VFA_mix_ system (54.5 ± 8.0 wt%) [64]. It can be concluded that substrate switching influences accumulation and enrichment with mixed VFAs leading to maximum yield. Integrating wastewater treatment with PHA copolymer production presents the advantage of repurposing environmental waste to generate an environmentally friendly end product however, the process needs to optimize the waste processing conditions and process parameters to attain the maximum conversion.

## Polyhydroxyalkanoates production utilizing wastewater resources

**Fig. 2:**
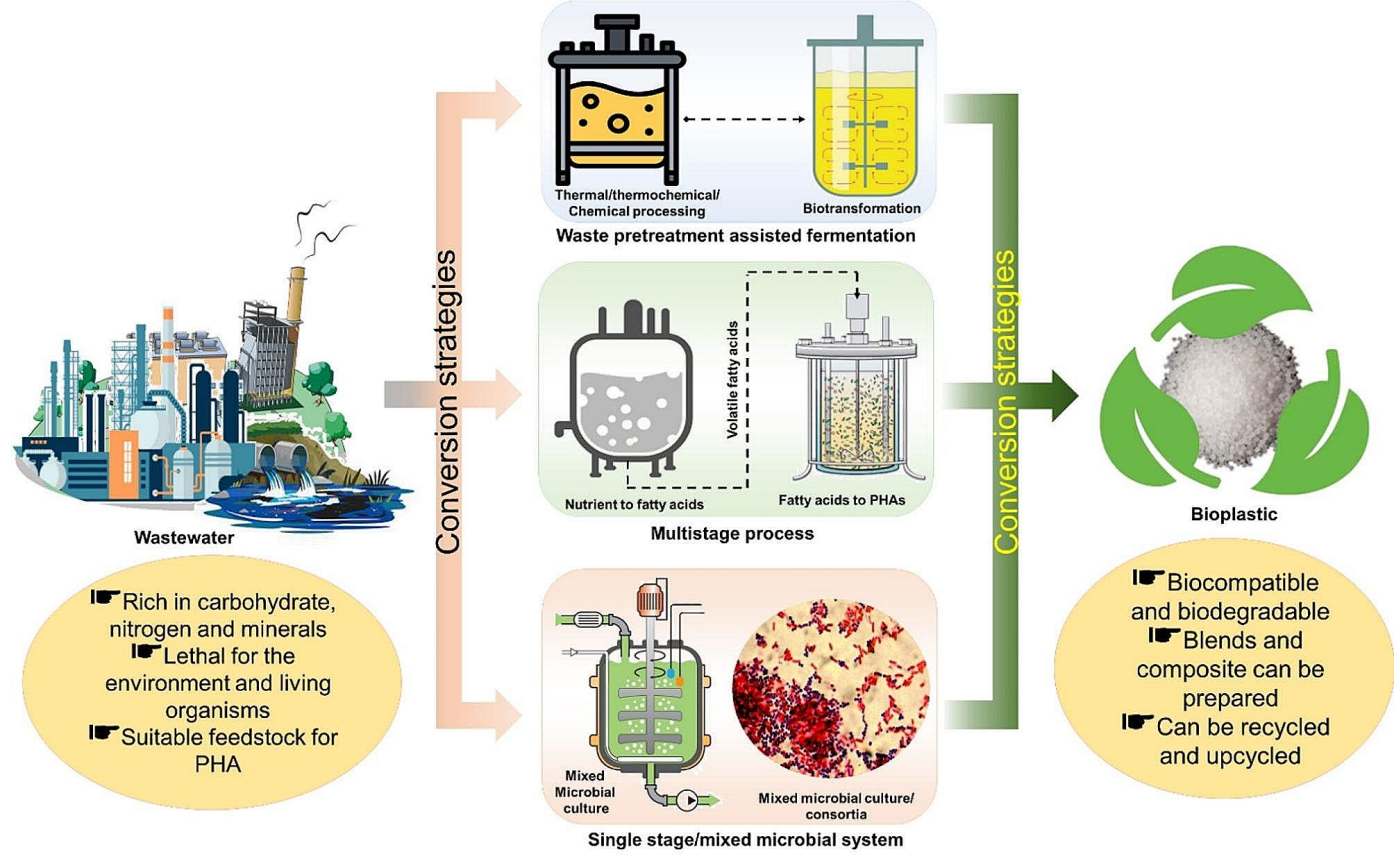
Different strategies for bioplastic production from wastewater

Polyhydroxyalkanoates constitute a class of biodegradable polymers synthesized by microorganisms as a means of intracellular carbon storage [65]. They present a sustainable and environmentally friendly alternative to traditional plastics and packaging materials. Certain bacteria have the capability to accumulate PHA from organic substrates, converting these carbon sources into valuable biopolymers [66, 67]. Recent studies have revealed a mutually beneficial relationship between wastewater treatment and PHA production, signaling a shift towards sustainable practices that leverage waste streams to produce biodegradable bioplastics, thereby promoting a circular economy [68]. The process of wastewater treatment, resource recovery, and conversion into PHA entails various benefits and challenges, as elucidated by recent research [69, 70]. To harness the nutrients available in wastewater for valorization, several strategies and approaches have been developed, as discussed below (Fig. 2).

### Wastewater composition and treatment challenges

Wastewater, sourced from domestic, industrial, and agricultural activities, presents a complex mixture of pollutants, both organic and inorganic, requiring sophisticated treatment methods to mitigate its adverse effects on ecosystems and human health [71, 72]. These compounds pose significant risks to lives and contribute to high oxygen demand [73]. Elevated levels of nutrients such as nitrogen and phosphorus, originating from human waste, detergents, and agricultural runoff, can induce eutrophication, leading to algal blooms and degradation of water quality [74].

Industrial effluents from textiles, pharmaceuticals, paper and pulp, and petroleum, etc., introduce a wide array of chemicals including heavy metals, pharmaceuticals, pesticides, and other toxic substances (Table 1), posing risks to ecosystems and human health [75, 76]. The paper and pulp industries rely on plant biomass as feedstock hence effluent [77] and granular sludge bed [78] from paper and pulp industries have high Chemical Oxygen Demand (COD), Total Dissolved Solids (TDS) and Total Suspended Solids (TSS) mainly contributed by phenols along with heavy metals. Asphalt reclamation and production sites generate highly toxic and polluted wastewater referred as Bitumin fume condensate wastewater which has shown high COD due to high organic load [79]. In contrast to food and paper industries, petrochemical industries effluent is also rich in COD, and total organic carbon (TOC) but the COD mainly contributes to processing waste like terephthalic acid, NH_3_-N, and volatile fatty acids [80].

**Table 1:** Wastewater from different industrial sources

Wastewater	Site	Main components and conductivity (EC)	Bio-chemical characteristics	References
Olive mill wastewater	-	Organic acids (formic and acetic acid), flavonoids, tannins, polyphenols	pH 2–6; high solid matter and organic load	[81]
Anaerobic digest of industrial wastewater from Candy production plant	InnovEn S.R.L. Turin, Italy	Lactic acid, propionic acid, acetic acid	TOC: 1.8% w/w	[82]
Season seed wastewater	Kasih Canned Food Production Co Amman (Jordan)	EC 2.5 ms.cm^− 1^	pH: 5.4; COD 3750 mg.L^− 1^_,_ NH_4_ 70 mg.L^− 1^, TS: 10,245 mg.L^− 1^	[83]
Dairy wastewater	-	EC 2.2 ± 0.1 ms	pH 8.62 ± 0.02, TSS: 265 ± 3 mg.L^− 1^_,_ TDS 934 ± 3 mg.L^− 1^, COD 1,999 ± 10 mg.L^− 1^, TS: 1,889 ± 4 mg.L^− 1^	[84]
Palm oil industries’ wastewater	-	EC 14.3 ± 0.1 ms	pH 2.05 ± 0.02, TSS: 518 ± 4 mg.L^− 1^_,_ TDS 9,889 ± 4 mg.L^− 1^, COD 1,596 ± 9 mg.L^− 1^, TS: 10,415 ± 5 mg.L^− 1^	[84]
Vegetable industry wastewater	-	Cadmium, nickel, lead, cobalt, *E. coli* 2336.67 CFU.g^− 1^_,_ EC 2.54 ms,	pH 6.90, VSS: 899 mg.L^− 1^_,_ TP 39.02 mg.L^− 1^, COD 5392 mg.L^− 1^, toxicity 29.167 Eqtox.m^− 3^¸ TSS: 1281 mg.L^− 1^	[85]
Domestic wastewater sludge	Wastewater treatment Palermo University Italy	-	TSS: 6.15 ± 1.22 mg.L^− 1^, VSS 4.35 ± 1.11 mg.L^− 1^, sCOD 133.59 ± 93.91 mg.L^− 1^, TCOD 6320 ± 2217.93 mg.L^− 1^	[86]
Cashew industry wastewater	-	Microbial load 4.8 × 10^6^ CFU.mL^− 1^, oil and grease 940 mg.L^− 1^	TSS: 551.43 mg.L^− 1^, pH 5.34, BOD 1161 mg.L^− 1^, COD 5740 mg.L^− 1^, total phenol 294 mg.L^− 1^	[87]
Winery wastewater (Vintage)	Niagara	Heavy metals including Hg 12,600 µg.L^− 1^, Cu 28,400 µg.L^− 1^, Mn 6100 µg.L^− 1^, Ni 3000 µg.L^− 1^, EC 1200–3400 µS.cm^− 1^	pH 4.24, COD 360,000 mg.L^− 1^, TSS: 70,300 mg.L^− 1^, BOD 96,000 mg.L^− 1^, TP 714 mg.L^− 1^, TVS 128,000 mg.L^− 1^	[88]
Winery wastewater (Non-vintage)	Niagara	Heavy metals including Hg 4170 µg.L^− 1^, Cu 29,600 µg.L^− 1^, Mn 7000 µg.L^− 1^, Ni 500 µg.L^− 1^, EC 355-12800 µS.cm^− 1^	pH 4.76, TSS: 84,400 mg.L^− 1^, COD 55,852 mg.L^− 1^, BOD 3160 mg.L^− 1^, TP 102 mg.L^− 1^, TVS 112,000 mg.L^− 1^	[88]
Petrochemical wastewater	-	Phenols 23 mg.L^− 1^, mercury, silver, arsenic, titanium, lead, copper, chromium, cadmium	pH 4.3–10.0, TOC: 1500 mg.L^− 1^, COD 1220 mg.L^− 1^, TSS 1.2–1000 mg.L^− 1^, NVFAs 275, VFAs 4900 mg.L^− 1^, TSS 1000 mg.L^− 1^	[89]
Raw winery wastewater	West Coast of South Africa	Turbidity 570–639 NTU, conductivity 175–230 mS.m^− 1^	pH 4-4.5, TSS: 1840–2275 mg.L^− 1^, TDS 1476–3546 mg.L^− 1^, COD 12,400–22,620 mg.L^− 1^	[90]
Petrochemical wastewater	-	Oil 500 mg.L^− 1^, salt 2157 mg.L^− 1^	pH 7.78, SS 200 mg.L^− 1^, CODcr 745.87 mg.L^− 1^, N_ammonia_ 40 mg.L^− 1^,TDS 2000 mg.L^− 1^	[91]

The release of such pollutants is mainly responsible for deteriorating the environment, therefore, the treatment of wastewater for the removal or degradation becomes mandatory [24, 92, 93]. Based on the nature of the treatment technique, wastewater treatment techniques are divided into physical, chemical, and biological treatments [94]. Physical treatment methods like screening, sedimentation, and filtration are employed to remove solid particles and larger pollutants but may not effectively eliminate dissolved substances. However, in chemical treatment, advanced oxidation processes and chemical coagulation aim to degrade or remove persistent pollutants. The methods are effective but also cost-intensive and generate secondary contaminants. In contrast, biological treatment by activated sludge, biofiltration, and constructed wetlands harness microbial activity to break down organic matter but may be less effective for certain pollutants [74, 95, 96]. The challenges and limitations of each wastewater treatment technique vary, not just in terms of initial capital and operational running costs, but also in terms of operatability, effectiveness, reliability, pre-treatment needs, environmental impact, and the generation of sludge and toxic by-product waste. Besides high and recalcitrant pollutant load and lack of standard processes, wastewater treatment facilities globally suffer from aging infrastructure, inadequate capacity, out dated technologies, limited funding, lack of skilled labor, and poor maintenance of treatment facilities. In addition to wastewater treatment, microbial metabolism also provides a chance to transform the organic nutrients in waste into high-valued commodity products like bioplastic and related precursors including PHA that not only lower the pollutant load but also provide an opportunity to generate revenue and employment that ultimately contribute to a circular economy.

### Strategies for wastewater-to-PHA conversion

The selection of suitable microbial strains, their metabolic capabilities, and their adaptation to the wastewater environment contribute to PHA production variability. The design and configuration of the employed bioreactor system also impact overall efficiency in wastewater treatment [97, 98]. On-going research focuses on advancing bioreactor design, optimizing microbial consortia, and exploring novel microbial species to improve wastewater-to-PHA conversion efficiencies [61, 99, 100]. The integration of wastewater treatment with PHA production embodies a sustainable approach aligned with circular economy principles, where waste is redefined as a valuable resource. Therefore, the optimization of the process and the adoption of various strategies become imperative. Some of these strategies include the Feast-Famine process, batch, and continuous-flow systems, the utilization of microbial consortia, co-feeding, and single and multistage or integrated processes, which are discussed below.

#### Feast-famine process

It represents a dynamic biological approach to wastewater treatment, embodying an innovative strategy that leverages the inherent resilience and adaptability of microbial communities to varying growth conditions, effectively eliminating pollutants and contaminants from wastewater sources. This approach has given rise to the evolution of a cyclic feast-famine regime, optimizing biological nutrient removal while reducing energy consumption and operational costs [101]. The Feast-Famine (F/F) process operates by alternating cycles of nutrient excess and limitation, primarily carbon and nitrogen or phosphorus, within wastewater treatment systems [102]. During feast periods, characterized by an abundance of nutrients, microbial growth, and substrate uptake are rapid, with microorganisms adapting to store excess nutrients. In contrast, famine periods induce the utilization of stored compounds and reserves for growth [103]. This cyclic F/F approach enhances biological nutrient removal efficiency, particularly for nitrogen and phosphorus, through mechanisms like denitrification and enhanced biological phosphorus removal.

The comparative assessment of F/F (0.2 and 0.6) revealed that biomass was able to accumulate higher PHB (approximately 500 mg.L^− 1^) at F/F 0.6 from acetate as feed while [103] for PHA production from VFAs, optimum F/F ratio was close to 0.2 d_feast_/d_famine_ [104, 105]. Similar observations were represented as maximum PHA accumulation (around 80%) was reported at F/F 0.2 [106]. It is noteworthy that the effect of F/F ratio on PHA accumulation may also be influenced by low dissolved oxygen (DO) concentrations. It was suggested that high DO becomes critical for biomass production while low DO supports PHA accumulation when butyrate and valerate are used as feed [107]. It is a general observation that DO becomes crucial for feed uptake by cells and hence there is a sudden spike in DO at the end of feast period. As per the reports, F/F affects PHA accumulation when DO is below 2 mg.L^− 1^ [108, 109] while there is no effect of F/F on PHA production if DO is higher than 3 2 mg.L^− 1^ [110, 111]. The adaptability of the F/F process makes it suitable for various wastewater types, including municipal wastewater, industrial effluents, and decentralized treatment systems. Maintaining stable microbial communities and preventing process upsets, such as bulking or foaming, remains a challenge that requires robust control strategies [112]. The F/F process typically requires less energy for aeration and promotes efficient nutrient removal, reducing operational costs in wastewater treatment plants [103]. Therefore, combining the F/F process with emerging technologies, such as bioinformatics, sensor advancements, and process automation, holds promise for enhanced efficiency and control [102, 103].

The utilization of the F/F strategy in PHA production may encounter challenges due to organic load variability [103]. Normally, the implementation of this culture strategy occurs in a Sequential Batch Reactor (SBR), where external substrates are alternated between excess and deficiency [113]. Typically, the operation cycles of SBRs follow fixed time intervals [106, 114]. Consequently, variations in operational conditions can lead to adjustments in the relationship between the Feast and Famine periods. For instance, an increase in organic load can extend the feast period, resulting in a shorter famine period if the total cycle time remains constant. Previous studies have demonstrated that changes in operational conditions, such as organic load rate and cycle time, significantly impact the performance of SBRs and PHA production [112, 115]. Therefore, it is evident that applying different organic loads would necessitate adjustments to feast and famine times. Based on the operation, the process can be classified into batch, fed-batch, and continuous operation. The comparative assessment of batch and fed-batch process with VFAs as a feed from synthetic and wastewater suggested that under the same operating conditions higher PHA content accumulation (%) was reported from fed-batch mode. The PHA yield was increased from 0.48 ± 0.006% (batch) to 0.52 ± 0.03% (fed-batch) in a much lower time [54].

#### Continuous flow system

Continuous-flow system is another approach to achieve high throughput bioproduction as well as waste treatment. It represents a progressive approach that streamlines and optimizes the processing of effluents with consistent and uninterrupted flow. In contrast to batch processes and fed-batch processes, it offers advantages in efficiency, stability, and adaptability, making them pivotal in addressing the ever-growing challenges of managing and treating wastewater [116]. Continuous-flow-activated sludge processes utilize microbial activity in aeration tanks to biologically treat wastewater, achieving efficient organic matter and nutrient removal [117]. Semi-continuous mode for PHA production from different types of sludge recovered from wastewater treatment plant yielded 28.4% in 24 h cycle at 20 ^o^C, neutral pH, and lower substrate concentration. Theoretical assessment of the process suggested that German wastewater treatment plants alone can compensate for 19% of global biopolymers production [118]. A substantial portion of the costs associated with PHA production involves creating sterile conditions, refining substrate carbon sources, using and disposing of solvents for polymer extraction, and dealing with low productivity, yields, and limitations such as short production campaigns and downtime during batch changeovers in batch/fed-batch operation modes [37, 119, 120]. To address these challenges, halophilic microorganisms have been proposed as cost-effective PHA producers. The high salinity required for halophiles to thrive in the production medium (150–200 g.L^− 1^ of salts) minimizes contamination risks, eliminating the need for sterile conditions [121]. This allows for open vessels and a low contamination risk continuous process [119, 122, 123]. Parroquin-Gonzalez and Winterburn [117] used a halophile *Haloferax mediterranei* for poly(3-hydroxybutyrate-*co*-3-hydroxyvalerate) production from VFAs in continuous mode and reported an increase in cell density from 0.29 - 0.38 mg.L^− 1^.h^− 1^ (fed-batch fermentations) to 0.87–1.43 mg.L^− 1^.h^− 1^ (continuous fermentation). Additionally, the downstream process can be significantly simplified by extracting the polymer through a straightforward osmotic shock when transferring cells into an isotonic media, thus eliminating the necessity for toxic and expensive solvents [124, 125].

#### Mixed microbial culture

Microbial consortia or mixed microbial culture (MMC) offers an advantage over monoculture as it allows the exploitation of synergistic dynamics, diverse metabolic capabilities, and pathways to deal with high organic load or complex pollutants present in wastewater [126]. The interactions among microorganisms, including mutualism, competition, predation, and syntrophy, drive the functionality and resilience of mixed cultures in various environments [127]. MMCs also become useful for PHA production from waste residues along with achieving maximum productivity, aiming for high mass polymer production per unit volume with high efficiency and lowered costs [128, 129]. Various studies have reported the production of PHA through MMCs involving *Proteobacteria, Bacteroidetes, Firmicutes, Acidobacteria*, *Candidatus, Saccharibacteria* from activated sludge substrates [130], *Plasticicumulans acidivorans*, and *Methylobacillus flagellates* from aerobic activated sludge and synthetic carbon source [131], *Alphaproteobacteria* and *Betaproteobacteria* from activated sludge and crude glycerol [109], and *Selenomonadales, Anaerobaculum*, and *Coprothermobacter* from raw sludge and thermally hydrolyzed sludge [36].

MMC also offers higher stability and functionality that can withstand environmental fluctuations and changing conditions [126]. Advancements in omics technologies and computational modeling offer insights into the intricate networks and metabolic interactions within mixed microbial communities [132]. Integrating mixed culture-based approaches with emerging technologies, such as artificial intelligence and high-throughput screening, holds promise for optimizing and scaling up biotechnological processes [133]. The limited competitiveness of PHA output compared to pure culture fermentation poses a significant challenge to the industrial scaling-up of the MMCs process [134, 135]. Nonetheless, an industrial-scale viable alternative lies in the PHA production process based on extended cultivation. For instance, Huang et al. [136] introduced an extended cultivation strategy for PHA-accumulation using MMCs. In their study, batch assays demonstrated high PHA content in cultivated MMCs, reaching 71.4% and 66.7% (higher than the 62.1% in the seed biomass) after 10 days of extended cultivation with and without sludge discharge, respectively. Incorporating this extended cultivation process achieved an overall PHA storage yield of 0.49 g COD PHA/g COD VFA and a volumetric productivity of 1.21 g PHA L^− 1^d^− 1^ with a final cell density of 17.22 gL^− 1^. Furthermore, the PHA accumulation ability was significantly enhanced by enrichment, irrespective of temperature and pH. Enrichment at 20–28 °C without pH control appeared most suitable for robust PHA accumulation [137]. Analysis of PHA accumulating microorganisms composition using the clone library method targeting *phaC* genes revealed that Burkholderiales dominated the seed sludge. However, after enrichment without pH control, Rhodocyclales, specifically *Azoarcus* spp. and *Thauera* spp., emerged as dominant, showcasing a robust ability to accumulate PHA.

#### Single and two-stage fermentation

The major application of the multistage process is the utilization of complex nature pollutants that obstruct their degradation [138]. In a single-stage process, PHA is generated directly from wastewater, whereas in a two-stage process, waste resources undergo initial fermentation into volatile fatty acids (VFAs) before being employed for PHA production. Municipal wastewater treatment plants (WWTPs) generate sludge as a by-product, and its effective management is crucial for controlling operating costs. Large WWTPs commonly employ anaerobic digestion (AD) to convert sludge into methane, reducing its mass. However, the current low market price of methane suggests an opportunity to explore alternative high-value products from sludge organic matter, such as PHA directly or via VFAs [139, 140]. Among the available wastewater, acidogenic effluents are a feasible option, as these can be derived from readily available regional sources of municipal and industrial organic wastewater and sludge. However, some waste has low concentrations of total VFAs i.e. 0.5 to 10 gL^− 1^ [126] while higher VFAs concentrations can be found in specific cases such as dairy [141] and fishing [142] industry residues, these may not provide a sufficiently large and widespread source for industrial-scale MMC PHA production. Colombo et al. [143]., valorized organic acids from municipal waste and yielded PHA production of 223 ± 28 g.kg^− 1^_total OA fed_. PHA, produced from organic fraction has a molecular weight of 8∙105 kDa and is comprised of hydroxybutyrate/hydroxyvalerate 53/47 (%). In another work, Ospina-Betancourth et al., [144] used yeast production industry wastewater (WWY) for the production of polyhydroxyalkanoates in sequential anaerobic reactors (reactor A) followed by two aerobic reactors (reactor B and C). VFAs produced in an anaerobic batch reactor (for 78 days), raw as well as distilled effluent from reactor A were used as feed PHA-production in reactors B and C (aerobic). The sequential process with mixed culture has yielded a maximum PHB accumulation of 17% of cell dry weight (1.2 g_PHB_.L^− 1^) from distilled effluent. Roche 454 16 S rRNA gene amplicon pyrosequencing identified *Paracoccus alcalophilus* (32%) and *Azoarcus* sp. (44%) as a dominant microbial population for PHB production. However, with widespread implementation and operation of the process, the availability of VFAs becomes crucial and hence waste collection, VFAs extraction, and processing are necessary parts of the process. In contrast to single stage process, two-stage processes compartmentalize operations into distinct stages or reactors, enabling a sequential or parallel execution of specific reactions or treatments and offering enhanced control over individual reaction kinetics or treatment conditions, enabling optimization of specific steps or facilitating intricate reactions [145].

#### Co-substrate feeding

The wastewater from different origins has a diverse composition which may become insufficient to maintain the stability of bioprocess. Hence use of multiple types of wastewater together might offer an advantage over monosubstrate systems [146]. The multisubstrate system would have higher productivity and stability due to nutrient balance, enhanced microbial metabolic diversity, and promotion of more comprehensive waste breakdown [147]. Usually waste from different industries has to be treated with chemicals or thermochemical treatments which encounters numerous technical and economic challenges related to product selectivity, conversion kinetics, yields, and potential applications. In comparison to chemical or thermochemical treatment, co-feeding, involving the simultaneous use of various feedstocks, offers synergistic benefits to enhance product yield and quality during the conversion process [148]. Valentino et al. [149]., combined municipal solid sludge and an organic fraction of municipal solid waste from the same urban area and used it for PHA production at a pilot scale using a three-step mixed microbial culture (MMC) process. Under optimum conditions, PHA specific storage capacity was 258 mg COD_PHA_.gCOD_Xa_.h^− 1^. PHA accumulation capacity of mixed culture via fed-batch process was 46 wt % PHA (dry cell weight) and offered an overall yield of 65 g_PHA_.Kg^− 1^_TVS_. Caproic acid is one of the intermediates for PHA production. Iglesias-Iglesias et al. [150]., have reported caproic acid production by co-digestion of cheese whey and sewage sludge. In a continuous mode of operation, maximum acidification of 44% was achieved at hydraulic retention times (HRT) of 10 days and 2 feeding cycles per day. Under optimum conditions, caproic acid rich stream resulted in PHA, copolymer of HB-*co*-HV-*co*-HHx. Owusu-Agyeman et al., [151] also reported the volatile fatty acid production from codigestion of sewage sludge and organic waste. An increase in organic load shift the VFAs composition towards caproic acid as a dominant proportion (> 55%). The major advantage of codigestion is the availability of nutrients by using a diverse range of waste as feed that possibly eliminates the shortcomings of individual wastewater however optimization of parameters is necessary to prevent the toxic or inhibitory effect of pollutants.

## Bioprocess parameter affect and scaleup studies

### Process parameter effect

The production of PHA from wastewater is a complex process influenced by various factors. One crucial factor is the composition of wastewater, with different organic substrates serving as feedstocks for PHA-producing microorganisms [45]. Calero et al., [152] compared organic loading rate (OLR) and VFAs production from cheese whey (estimated via the degree of acidification (DA)) via up-flow anaerobic sludge blanket reactor (UASB; continuous process) and sequencing batch reactor (SBR; discontinuous process). Both reactors have a maximum DA of 98% with an OLR of 2.7 g_COD_.L^− 1^.d^− 1^ in SBR and 97% with an OLR of 15.1 g_COD_.L^− 1^.d^− 1^ in UASB. It is distinct that continuous process would be able to handle higher OLR while in both cases product was mainly dominated by acetate, butyrate, propionate, and valerate. The availability and concentration of these substrates, such as VFAs and sugars, play a pivotal role in determining microbial growth and subsequent PHA accumulation. As per the literature, VFAs are the favorable substrate in comparison to sugar or other organic molecules for PHA production. Another observation suggested that not only VFAs amount but also composition has a direct effect on the productivity as well as composition of PHA [153]. A study revealed that mixed VFAs have higher productivity in comparison to monosubstrate systems. In context to composition, butyrate-rich VFAs feed has a maximum PHA accumulation of 72.08% of VSS followed by valerate-rich feed (61.57%). However, the bioplastic produced was more robust in the case of valerate-rich feed and was mainly comprised of 3-hydroxyvalerate (HV) (more than 20%) [154]. Further, the presence of HV in PHA increased in average molecular weight and crystallinity [155]. The concentration of HV in PHA might be responsible for the amorphous nature of bioplastic as its increasing concentration lowers the crystallinity and melting temperature and makes it sensitive to thermal degradation. VFAs stream produced from chicken manure (VFA_CM_) and potato peels (VFA_PP_) have been compared for PHA production and composition based on feed composition. VFA_PP_ is rich in acetic acid, and ammonium nitrogen while VFA_CM_ was rich in butyric acid, and valeric acid. The maximum bioplastic production was reported from potato peels but comparative analysis revealed that PHA from chicken manure has lower volatile mass and higher dehydration temperatures while PHA from VFA_PP_ has higher thermal degradation temperature [156].

Nitrogen and phosphorus concentration also play an important role in PHA accumulation. To study the C/N ratio’s effect on PHA production, Valencia et al. supplemented activated sludge with acetate and ammonia in a sequencing batch reactor (SBR) to maintain C/N (13.3–42.1) and found C/N of 23.3 results in higher PHA accumulation, and beyond this, there is negative effect [157]. Zhang et al., studied PHA accumulation from activated sludge at pH (7.5 ~ 8.5) with various C/N and C/P ratios and observed 150 as the optimum ratio for maximum PHA accumulation i.e. 50.39% and 36.07% respectively [158]. Tu et al., [159] studied the phosphorus limitation effect on PHA accumulation from thermal hydrolyzed sludge and reported an increase in PHA content from 23 to 51% when phosphorus concentration decreased from 127.5 to 1.35 mgL^− 1^.

Furthermore, the operational conditions of the wastewater treatment process, including temperature, pH, and dissolved oxygen levels, influence microbial activity and metabolic pathways leading to PHA synthesis. Temperature has a direct influence on biomass hydrolysis and results in increased microbial biomass production which ultimately leads to increased PHA accumulation. The availability of acetate and temperature also affected the microbial diversity as in the beginning sludge was denoted with 29 species which was reduced to 16 after community selection. Mostly non-defined genera were eliminated and post-selection, the community composition was represented by Mucilaginibacter. The reason for elimination was also the unavailability of nutrients to non-PHA accumulating microorganisms. In contrast, De Grazia et al., [160] used mixed microbial culture that was much more stable and tolerant and hence was able to accumulate around 60–65% g_PHA_.g_VSS_^−1^ from acetic acid at 15–25 ^o^C which supported the fact that mixed microbial culture was stable in seasonal variation.

Availability of oxygen becomes critical for microbial cell growth as well as PHA production and characteristics. Available oxygen also affects the pollutant’s removal from wastewater especially in the context of nitrification. At higher oxygen availability, both PHA production and nitrification occur in the reactor while in reduced dissolved oxygen (DO) nitrification process is halted and only PHA production occurs. However, high DO levels support PHA accumulation and acetate, butyrate, propionate, and valerate as dominant VFAs while at lower DO acetate and propionate dominate [107, 161]. At lower DO and bioreactor oxygen transfer rate production of short-chain length PHA induced [162].

### Scale-up studies

Maximum bioplastic and intermediates like VFAs production from wastewater can be achieved under optimum growth conditions but commercial application of bioplastic needs to scale up with maximum resource utilization along with maintenance of bioplastic yield. However, some of the researchers have shown the sincerest effort to scale up the PHA production to pilot or industrial scale from industrial wastewater at high organic load (mostly fat and lipids). At the laboratory scale, *Ralstonia eutropha* proved to be an efficient PHA producer from glucose and fructose while *Bacillus megaterium* was better in the case of whey as feed [163]. The overall assessment led to the selection of *R. eutropha* for upscaling the PHA production at 3 L working volume. At 2 L production scale, *R. eutropha* attained a maximum PHA content of 4.19 gL^− 1^ (74.2%) with substrate consumption of 79.0%. The overall process has a PHA yield of 0.72 (YP/x) and productivity of 0.19 g.L^− 1^.h^− 1^ [164]. PHA production from sewage sludge at a continuous stirred tank pilot scale bioreactor (225 L) showed that domestic sewage sludge can be a good feed as a PHA yield of 0.37 g_PHA_.g_VFAs_ was obtained even with low organic loading of 0.06 Kg_BOD_.Kg_SS_^−1^.day^− 1^ [86].

The major issues with wastewater-based PHA production at pilot and industrial scales were operating conditions, the composition of wastewater, salt concentration, and pH of the medium. The seasonal variation in wastewater has a major influence on PHA production as VFAs are the prime source for PHA production. In the case of lower concentration of VFAs in wastewater, non-PHA accumulating microorganisms biomass increased while PHA accumulating and VFAs consuming microbial diversity reduced. Hence lower VFAs (0.35–1.00 g_VFA−COD_/gs_COD_) was not preferred [165]. The influence of temperature on the enrichment of biomass and PHA production by activated sludge was evaluated within a practical case study. Two laboratory-scale sequencing batch reactors (SBRs) were operated at different temperatures (15 and 25 °C) in parallel over 131 days to treat wastewater from a potato-starch modification facility and produce surplus activated sludge biomass with PHA accumulation potential. Temperature did not influence wastewater treatment performance (average 97% COD removal). Several other researchers have summarised the efforts for PHA production from wastewater from different sources at various operational scales are summarised in Table 2.

**Table 2:** Polyhydroxyalkanoates production from different substrates

Organisms	Source	Scale	Process conditions	Major outcome	PHA yield % w/w	Productivity g.L^− 1^	References
*Synechocystis* MT_a24	Urban wastewater	100 L	Thin-layer raceway pond; light intensity 100–800 µ mol photons m^− 2^.s^− 1^; pH 7.5-8.0; night temp 18–22 ^o^C and morning 27–39 ^o^C.	Maximum PHB was at a late stationary phase (under nutrient-limited conditions) *Poterioochromonas malhamensis* contamination was eliminated by *Synechocystis* cultivation in an alkaline environment.	23.7 ± 2.2	0.62	[166]
Aerobic granular sludge	Epichlorohydrin wastewater	1 L	Batch process; working volume 1 L; temp 25 ^o^C; aeration 1.5 L.min^− 1^.	COD removal efficiency 70%; sludge dominated by *Halomonas* sp. (86 ± 0.50%).	52.67	-	[167]
*Nordic* sp.	Wastewater	250 mL	Temp 30 °C; mixing rate 180 rpm; production 72 h; pH 7.0.	Wastewater pretreated by thermochemical treatment with 1% H_2_SO_4_ increases the solubility of total sugars.	27	3.83 ± 0.24	[168]
*Synechocystis* sp.	Secondary effluent	10 L	pH 7.99; conductivity 2.47 ± 0.08 mS.cm^− 1^; COD 96.65 ± 40.70 mgO_2_.L^− 1^	Hydraulic retention time (HRT) 8 d; lipid accumulation 44.7% (30 d)	4.8	-	[169]
*Bacillus* sp. CYR1	Cheese whey + raw sewage (Rantoh wastewater treatment plant, Japan)	500 mL	4% *Bacillus* sp. CYR1 inoculum; 30 °C and 120 rpm for 96 h.	Cell mass of 692.4 ± 7.62 mg.L^− 1^_,_ sewage has low organic content hence addition of high organic carbon is needed for PHA accumulation.	24.1 ± 0.70	0.147	[170]
Bacterial isolate	Cashew industry wastewater	250 mL	Dried microbial biomass suspended in 4% sodium hypochlorite solution and hydrolysis was conducted at 37 °C for 1 h. PHA was extracted from a cell pallet with chloroform	Maximum PHA accumulation was 34.04% with 20% wastewater. An increase in wastewater beyond this, reduced PHA accumulation. Phenol can also be used as a carbon source	34.04	0.32	[87]
*P. aminovorans*	Lactic acid fermentation broth, swine manure, and apple waste	10 L	Temp 35 ± 1 °C and time 10 days; mixing rate 100 rpm and aeration 1 mL.min^− 1^	PHA content increased with a reduction in lactic acid concentration in the medium. At higher lactic acid concentrations, cells have competitive interaction and available carbon source used for proliferation rather than PHA	55.4	1.04	[171]
Waste activated sludge	Bath wastewater treatment plant (Rilland-Bath, Netherlands)	167 L	Temp 25 °C; mixing rate 230 rpm and aeration rate 50 Lmin^− 1^.	No PHA accumulation was detected in fresh activated sludge. Nile Red has better PHA accumulation signal generation than Nile Blue A	48 ± 2	-	[172]
*Bacillus pumilus* NMG5 strain	Paper factories wastewater (Minh Hung paper factory)	30 L	Dissolved oxygen 4 mg.L^− 1^	COD removal was 95.93%. Phosphorus and nitrogen removal efficiencies were 83.55% and 79.36%	42.28	-	[173]
*Haloferax mediterranei*	Sesame seed wastewater	5 L	Mixing rate 230 rpm, at 37 °C for 4 d, pH 7.0, aeration 0.75 VVM	6 g.L^− 1^ yeast extract and 100 g.L^− 1^ NaCl was optimum for PHA accumulation.	75	0.53	[83]
Mixed inoculum	Wastewater	10 L	Volatile suspended solids 100 mg L^− 1^; Temp 24 ± 4 °C; dissolved oxygen < 0.2 mg L^− 1^; pH 7 and time 5 days	Inorganic acid inactivation of metabolism increased the yield from 20–33%	35–38	-	[174]
*Pseudomonas mendocina* CH50	Waste frying oil	14 L	Temp 30 °C, mixing rate 200 rpm, air flow 1 vvm, and time 48 h	Biomass accumulation 1.03 g.L^− 1^ AuNPs/PHA composite reduced viral infectivity against SARS-CoV-2 pseudotype virus by 75%.	^−^	0.23	[175]
Microalgae	Aquaculture effluent	22 L	pH 6.8; period 14 d;	The high light intensity increased PHB accumulation while biomass yield lowered; no effect of light on lipid content; low-intensity red light was carbon neutral.	0.014	-	[176]
*Haloferax mediterranei*	Ricotta Cheese exhausted whey	3 L	pH 7.2, temperature 37 °C for 24 h and mixing rate 150 rpm, inoculum 20% (v/v)	The melting temperature was 146.2 ^o^C lower than commercial PHBV.	9.36	1.18 ± 0.06	[177]
*Cupriavidus necator* DSM 13,513	Dairy wastewater	4 L	Fed-batch fermentation, temp 30 °C, for 48 h, mixing rate 200 rpm	Yield was optimum in 12 h with aeration both in batch and fed bath mode. Accumulation of acetyl CoA induces the PHB synthesis.	0.52	-	[54]
Mixed microbial culture	Rice winery wastewater	-	Batch experiment DO > 2 mg.L^− 1^, pH 7 temperature 25–30 ^o^C	Maximum PHA yield at OLR of 2.4 g_COD_.L^− 1^.D^− 1^ *Zoogloea* was the most dominant PHA-accumulating organism.	23	135.6	[178]

## Advances in PHA recovery and purification

**Fig. 3:**
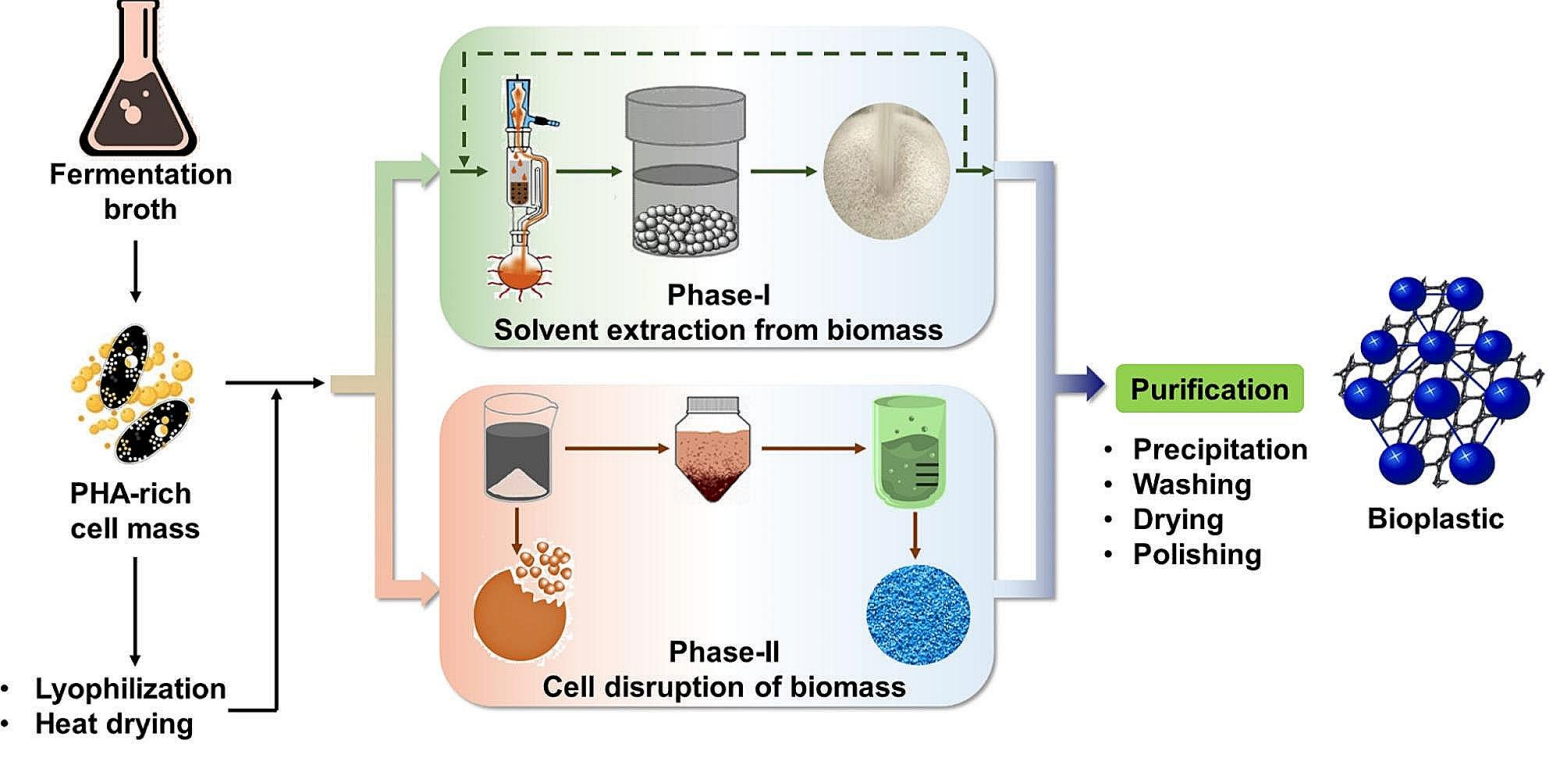
Downstream processing and product recovery of polyhydroxyalkanoates

PHA recovery and purification are also one of the challenges as it alone accounts for 30–50% of the total costs [179] and determines the process feasibility for industrial applicability. The product recovery underwent five crucial stages including biomass recovery/harvesting, pre-treatment of biomass, PHA recovery, and formulation [37]. Several strategies including solvent extraction, and microbial cell disruption have been suggested (Fig. 3).

The PHA recovery is a multi-step process that begins with cell mass separation followed by extraction or cell lysis. For PHA recovery, cell lysis can be achieved by physical, chemical, or enzymatic methods. Physical methods employ mechanical and shear forces like crushing and bead mills-based treatment for cell disruption [180]. In comparison, chemical methods use different chemical agents to degrade or depolymerize the cell coverings and expose the inner chamber. The methods have respective pros and cons like physical methods need high energy input and have questionable efficiency on a commercial scale. While chemical methods involve the use of toxic chemicals and generate inhibitory by-products e.g. furans and phenolics. Hence selection of a treatment strategy becomes an important aspect of downstream processing.

It has also been observed that integrated and combined treatment of physical and chemical approaches improves the cell lysis and recovery rate. Pillai et al., [181] have combined microwave treatment with EDTA followed by CHCl_3_ and NaOCl-assisted cell lysis for PHA recovery. The use of microwave-EDTA treatment has improved the molecular weight of the recovered polymer by 2.9 folds with 93.75% recovery and 97.21% purity. Another study also showed that microwave-based extraction has a higher rate of energy transfer in comparison to heat which improved the recovery rate in a lower time [182]. In comparison to physical cell lysis-based methods, solvent-based methods have higher applicability due to their ability to handle large volumes and can be used at an industrial scale and the possible recyclability of solvents that reduce the process cost. Solvent-based extraction of bioplastic and associated polymers includes treatment of microbial biomass with solvent or extraction agents followed by heating to the optimized temperature which is commonly below 200 ^o^C (usually below PHA decomposition) that solubilizes PHA in the organic phase and removes non-PHA mass in the aqueous phase. PHA from the solvent phase can be recovered by evaporation-facilitated distillation or filtration or using antisolvents to precipitate the PHA [38]. Solvents systems can be categorized based on the solvent nature (Table 3).

**Table 3:** Different solvent systems used for solvent extraction

Categories	Example	Advantage	Disadvantage
Halogenated solvents	Chloroform, dichloromethane, 1,2-dichloroethane, 1,1,2-trichloroethane, and 1,1,2,2-tetrachloroethane.	High molecular weight PHA (1.2 MDa) can be extracted. Extracted PHA will be of high purity.	High risk of environmental pollution.
Alkanes	Heptane, octane, and hexadecane.	Recovered PHA has a high purity.	Low yield of PHA. Polymer degradation during recovery.
Alcohols	Methanol, ethanol, n-propanol, iso-propanol	Short-medium chain polymer can be recovered.	Too polar for high-length polymers hence recovery will be low.
Esters	Ethylic esters	Purity will be high	Operated at high temperatures (90–100 ^o^C)
Carbonates	Diethyl carbonate, propylene carbonate, and ethyl acetate	Eco-friendly alternatives have minimal toxicity, and biodegradable	Low stability to temperature.
Ketones	Methyl ethyl ketone	Suitable for all temperatures and pressure.	Problematic and may be toxic.
Biobased solvents	2-methyltetrahydroxyfuran and dihydrolevoglucosenone (cyrene)	Non-toxic, greener, and sustainable.	Long extraction times and low extraction selectivity and yield.

As suggested in the table, each type of solvent system has its respective advantages and disadvantages. Chloroform is the standard solvent used for PHA recovery which is under chlorinated solvent that may have a toxic impact on recovered product thus non-chlorinated and green solvents are preferred. Comparative evaluation of non-chlorinated solvents (cyclohexanone and γ-butyrolactone) showed higher extraction efficiency (> 95%) with cyclohexanone in comparison to γ-butyrolactone [189]. Extraction conditions for solid loading, temperature, and appropriate solvent are crucial for product recovery. It has been observed that extraction at higher temperatures, has a higher recovery yield. Vermeed et al., [190] selected 6 solvents including 1-butanol, 2-butanol, 2-ethyl hexanol, dimethyl carbonate (DMC), methyl isobutyl ketone (MIBK), and acetone from 35 solvents based on toxicity to extract poly 3-hydroxybutyrate-*co*-3-hydroxyvalerate (PHBV), produced from organic waste streams using mixed microbial communities at pilot scale. Among the selected 6 solvents, acetone and DMC have offered a maximum yield of 91–95% with 93–96% purity. The selection of solvents also becomes critical due to the differential solubility of PHA followed by the removal and recycling of solvents. Over the conventional solvent system, the use of solvent-antisolvents system has shown higher efficiency as well as PHA purity. Mongili et al., [191] compared two solvent/antisolvent systems comprised of DMC/ethanol and chloroform/hexane for the extraction of PHB from wet and dry *Escherichia coli* biomass. In comparison to dry biomass, PHB yield was stable with DMC as well as chloroform-based systems while crystallinity was higher with dry biomass (53%) even higher than chloroform-based extraction (41%). The system has shown commendable properties however, the complexity of product recovery increased due to the use of multiple solvents.

Advanced extraction systems and the use of non-ionic surfactants and biobased solvents have represented greener and more cost-effective alternatives to conventional solvents for PHA extraction. Comparative evaluation between non-ionic surfactants (Tween® 20, Brij® L4, and Triton™ X-114) with DMC and CHCl_3_ have shown maximum yield with CHCl_3_ (63%) followed by Tween® 20 (50%) while the purity of extracted polymer was higher than 90% [179]. In another work, Elhami et al., [39] recovered around 62 ± 3% 3-hydroxybutyrate-*co*-3-hydroxyvalerate (PHBV) with > 99% purity from mixed microbial culture (grown in wastewater) with methyltetrahydroxyfuran (2-MTHF) at 80 °C in 1 h. Table 4 summarises major outcomes for PHAs recovery using different techniques.

**Table 4:** Downstream processing and recovery of PHA from microbial biomass at different scales

Source	Scale	Extraction conditions	PHA recovery %	PHA purity%	Molecular weight	Major outcome	References
Activate sludge	3 L	Supercritical CO_2_, pressure 200 bar, time 15 min at 40 °C.	80%	80%	0.27 × 10^6^	Biomass density of 57 g.L^− 1^. The presence of methanol as a modifier increased the molecular weight of the polymer.	[183]
Organic waste (Treviso plant)	380 L	Wet biomass acidified with H_2_SO_4_ centrifuged and undergoes aqueous phase extraction with inorganic reagents	-	98.9 ± 0.7%	424.8 ± 20.6 KDa	PHA contains 3-hydroxybutyrate (3HB) and 3-hydroxyvalerate (3HV) (92.6–79.8 and 7.4–20.2 w/w)	[184]
Lisbon plants	100 L		-	98.9 ± 0.7% w/w	224.9 ± 21.9 KDa	PHA contains 3-hydroxybutyrate (3HB) and 3-hydroxyvalerate (3HV) (92.6–79.8 and 7.4–20.2 w/w relative percentage)	
Carbonera plants	2800 L	Dried biomass undergoes aqueous phase extraction with inorganic reagents.	-	85.5 ± 3.6% w/w	424.8 ± 20.6 KDa	PHA contains 3-hydroxybutyrate (3HB) and 3-hydroxyvalerate (3HV) (44–13 and 56–87 w/w relative percentage).	
Fermented sewage sludge	100 L	Temp 115 °C and pressure around 300 kPa	70%	92%	571 KDa	NaOH + H_2_O_2_-based extraction has the highest recovery. It facilitates hydrolysis and oxidation.	[185]
Wastewater	10 L	Inactivation with 0.2% formaldehyde then cell mass recovery and lyophilization and extraction with chloroform.	20%	-	-	Comparatively economic and can be used at a higher scale as well accumulation was 33% dry cell weight.	[174]
PHARIO project activated sludge	-	Extraction with 10 mL dimethyl carbonate at 125 ^o^C for 60 min.	100%	95 ± 2	-	Thermal treatment of dry PHA-rich biomass lowers PHA molecular weight predictably.	[186]
Microbial sludge (food pulp waste feed)	60–100 L	Biomass dried and treated with 0.3 M NaOH at 30 ^o^C for 300 min	100	99	-	Standard protocol with CHCl_3_ has recovery of 81–83% recovery The purity of the final product increased with intracellular concentration and irrespective of extraction agent.	[187]
	60–100 L	Biomass dried and treated with 9.0% NaClO at 30 ^o^C for 3.4 h	90	100	-		
Secondary sedimentation tank (STP-300 KLD) Jawaharlal Nehru University, India	500 mL	2 g sludge biomass 200 mL CHCl_3_ at 70 ^o^C, 180 rpm for 72 h with continuous agitation.	8.3	96		90% 3-hydroxy-butyrate and 10% 3-hydroxy-valerate.	[188]

Process economics and energy investment also suggested solvent-based extraction as a low-energy operation and cost-effective approach. In terms of energy investment, the heat capacity of solvents/biomass is usually lying around 1–2 kJ.Kg^− 1^ K^− 1^ while the heat capacity of water is 4.2 kJ.Kg^− 1^ K^− 1^ (microbial slurry with 10–20 wt %). As the required energy for extraction operation is normally around 0.5–2 MJ.Kg^− 1^ PHA which showed the process is low energy intensive [38]. In comparison to conventional solvents, biobased solvents have superiority due to cost-effectiveness and low carbon footprint [179]. Besides, operational feasibility, solvent recyclability can further improve the economics and process life cycle as the same solvent can be used multiple times and reduces the residues and by-products generation from the process. Moreover, standardization of the extraction process becomes easier and makes it possible to adopt at an industrial scale as well.

## Sustainability considerations and techno-economic analysis

Plastic pollution has become a serious concern on global platforms and raised the alarm for future sustainability. The United Nations has put the concern related to plastic pollution in front of 187 UN members during the 73^rd^ session (2018–2019) and suggested the requirement for a transparent and regulatory framework besides including plastic waste in global hazardous materials with the amendment of the directives of the 1989 Basel Convention [192]. Along with this the UN Industrial Development Organization and G20 nations imposed a ban on plastic materials and ensured widespread participation in waste management by providing financial incentives [193]. Biopolymers have shown possible routes to reduce or eliminate petroleum-based plastic. Furthermore, the use of microbial fermentation using waste resources as feedstocks lowers the production cost of biopolymers. National and international government bodies are also emphasizing the shift to circular economy principles and utilization of waste resources to maintain sustainability.

The World Economic Forum, McKinsey & Company, and Ellen MacArthur Foundation proposed some initiatives like EPR (Extended producer responsibility) schemes to reduce the ocean leakage rates by 80% by 2040. The strategies include the prevention of waste exports into countries having high leakage rates, and improving the waste recycling capacity from 21 to 54% apart from eliminating the major microplastic sources. The European Union (EU) has also suggested policies framework to amend the European Green Deal and Circular Economy Action Plan. These suggestions include recycling around 50% of packaging plastic by 2030. As a result, the use of single-use plastic items like polystyrene-based beverage containers, cutlery, food and straws, cotton bud sticks, and all oxo-degradable plastics have been prohibited in the EU since January 2021 along with restricted export of low-grade plastic outside EU borders (as mentioned in Basel agreements). As a financial restriction, a high amount of tax (around €800 per tonne) has also been imposed on non-recycled plastic to suppress the use of non-recyclable plastic and to promote industries manufacturing eco-friendly, recyclable, biodegradable, and reusable plastic alternatives [194].

As per the report published, China is the largest producer of single-use plastics across the globe. With international commitments, China also announced the ban on the production of non-recyclables plastic by 2025 and shifted to degradable bioplastic [195]. To achieve the planned target, Chinese manufacturers have boosted PLA production to 700,000 tons per year and target the total outcome of polybutylene adipate-*co*-terephthalate (PBAT) and polybutylene succinate (PBS) to 1.24 million tonnes per year by 2023. On a similar track, other countries including Japan, Malaysia, Singapore, and South Korea have also proposed financial subsidies for bioplastics production [3, 194]. The main obstacle to the implementation of bioplastic as the main alternative to plastic at the global level is the associated cost. The assessments have been conducted to evaluate the production of different types of bioplastics under different environments and scales. Waste materials and resources are the prime feedstock for the cost-effective production of various products. Rajendran and Han [196] used food waste as low-cost raw material for poly (butylene succinate) (PBS) production and economic feasibility was assessed. The process suggested the minimum selling price (MSP) of PBS, produced from food waste was 3.5 $ Kg^− 1^ (determined by the Monte Carlo simulation). The process offered the plant’s return on investment (ROI) of 15.79%, with a payback period, and internal rate of return (IRR) of 6.33 years and 16.48% respectively. The process has a net present value of 58,879,000 USD. The analysis also revealed GHG emission of 5.19 Kg CO_2_eq Kg^− 1^ which was much lower than the conventional production process for PBS. PHA production from molasses by mixed microbial culture was evaluated which showed the PHA manufacturing process cost $994,143 with an annual process operation cost of $159,711 and a payback period of 6.79 years. The process has an internal return rate of 16%. It was also suggested that the benefit from the process could be increased by 25% if the product costs were reduced by 20% [197]. The process seems greener in terms of GHG emission while process parameters optimization and byproduct re-valorization might reduce the process cost. Besides food waste, other waste including agricultural residues process byproducts, and wastewater from residential as well as industrial areas can also be used as feed.

A simulation study for poly(3-hydroxybutyrate-*co*-3-hydroxyvalerate) production from cheese whey by a halophile ‘*Haloferax mediterranei’* was conducted by SuperPro Designer to assess the material flow and process economics. A local cheese plant was selected for the study that has around 168.7 metric tons of lactose.day^− 1^ (MT.d^− 1^) and conversion process was divided into three scenarios i.e. (a) without recycling/reuse of salt and enzyme, (b) enzyme reuse + non-recycling of salts, and (c) recycling and reuse of salts and enzyme. The recovered whey stream was reused for poly(3-hydroxybutyrate-*co*-3-hydroxyvalerate) production by halophiles. The simulation showed the production of 9700 MT_PHBV_ year^− 1^ (0.2 g_PHBV_.g_lactose_^−1^) with a conversion efficiency of 87%. The breakeven price for the product was sensitive to enzyme and lactose prices as it offered the lowest price of 4 $.Kg^− 1^_PHA_ [198]. Another analysis with sewage sludge from municipal wastewater treatment plants for bioplastic production at two different scales and processes i.e. (a) sludge is dewatered only and small operation scale (small wastewater treatment plant) and (b) anaerobic digestion of sewage sludge and large operation scale (large wastewater treatment plant). PHA production was conducted in a two-stage process which offered a minimum PHA cost of 1.26 US$.Kg^− 1^_crudePHA_ (large wastewater treatment plant) and 2.26 US$.Kg^− 1^_crudePHA_ (small wastewater treatment plant). In another scenario, a single-stage process was also studied in which secondary sludge was used for PHA accumulation. In this case, PHA production costs were further reduced by 19.0% and 15.9% for large and small wastewater treatment plants respectively which was mainly due to lowered capital investment [100]. Instead of conventional heterotrophic systems with bacteria, fungi, algae, and cyanobacterial systems have higher efficiency due to lower landscape and resource requirements. It allows the use of inorganic carbon from air (CO and CO_2_) as well as organic carbon from wastewater. Process economics for PHBs production from wastewater revealed that the minimum selling price of PHB was around 135 € Kg_PHB_^−1^ when PHB productivity was 12.5 g_PHB_ m^− 3^ d^− 1^ which makes around 50% of dry cell weight (dcw). However, productivity must be much higher (810 mg L^− 1^ d^− 1^) to compete with the market cost (i.e. 4 € Kg_PHB_^−1^) [199] which emphasizes a more in-depth analysis of the process and investment of resources. The analysis not only identified wastewater as potential feed but also reported chemical oxygen demand and operation size as critical factors. In addition, supportive governmental policies and technical upgradation seem mandatory to reduce the process cost and wide acceptability of biopolymers as an alternative to petroleum-based plastic.

## Future perspectives and challenges

In the past, polymers produced from petroleum have been extensively utilized in several applications, including textile, medical, transportation, chemical manufacturing, optical, and electrical devices. Nevertheless, the increasing demand for biopolymers can be attributed to various factors, including the volatility of oil prices, the environmental concerns associated with petroleum-derived biopolymers, advancements in biopolymer production technology, and the rapid development of biopolymer-based products. However, at present, bioplastics account for merely 1% of the total yearly plastic production [200]. The handfuls of start-ups producing bioplastic on a commercial scale are PHAXTEC, Inc. (Wake Forest, NC, USA), VEnvirotech Biotechnology SL (Barcelona, Spain), Verde Bioresins, Inc™ (Fullerton, CA, USA), Bhagirath Industries Private Ltd. (Gujarat, India) and Tianjin Green Bioscience Co., Ltd. (Tianjin, China).

The primary factor contributing to the low efficacy of bioplastic is its significantly higher production costs (2.2 to 5.0 €/kg), which are about three times greater than those of traditional synthetic plastics (less than 1.0 €/kg) such as polyethylene (PE) and polypropylene (PP) [99]. To compete with a petroleum-based plastic, it is necessary to address certain challenges, including the sustainable production of biopolymers. This involves overcoming issues such as the cost of production, scaling of the production process, and downstream of the products. The production cost can be reduced by up to 50% by considering locally available waste or residues as feedstocks for bioplastic production [201]. However, the variability of wastewater composition is contingent upon the source of feedstocks. Eventually, the variability in the efficiency of PHA production is observed. This issue can be solved to some extent by classifying and separating different wastes used as feedstocks for PHA production processes. Furthermore, wastewater is a complex substrate for microbial growth because it contains many different constituents, including nutrients and micropollutants. Therefore, strategies like acclimatization for extreme conditions, consortium, and multistage integrated processes have proved advantageous over pure culture for the efficient consumption of waste streams. In addition to that, naturally developed mixed culture reduces the cost of the process associated with sterilization [202]. In addition to high production cost, GHG emissions during production, use of complete degradation (complex polymers and composites), detailed analysis of negative ecological impacts (if traces remained in the system), unawareness of society, and insignificant resistance to water and hydrophilic environment are the other issues that affect bioplastic performance [203, 204].

Alternatively, genetic modification of PHA-producing microbes using CRISPR and Cas9 technologies could be promising for enhanced bioplastic production [204, 205]. For marketing, effective strategies need to be planned and implicated to enhance the adaptation of bioplastic by lower economy zones along with higher economy and middle economy zones [206]. Some of the recent works have shown the possible use of natural biopolymers from plants as plasticizers and reinforcement materials. It has been reported that the blending of starch lowered the use of non-renewable energy and GHG emissions by 60% and 80% respectively along with a 40% reduction in eutrophication potential 60% reduction in land use [203]. The research and marketing area have lots of opportunities as well as challenges that need continuous efforts for quick and efficient addressal. Moreover, alone replacement of petroleum-based plastic materials with bioplastic is not sufficient, and need to find some strategies to reuse plastic-based waste, accumulated in the environment.

## Conclusion

Polyhydroxyalkanoates (PHA), a biodegradable polymer, presents a promising alternative to traditional plastics, offering potential solutions for reducing packaging waste. The wastewater generated by industrial, agri-horticultural, and municipal activities is rich in organic and inorganic compounds, including carbon, nitrogen, phosphorus, and minerals, serving as a natural resource for microorganisms to produce PHA. The selection of microbes through methods like the feast and famine approach, along with the utilization of microbial consortia, enhances the efficiency of PHA production processes. However, challenges persist in the downstream processing of PHA, as existing extraction methods often yield lower quantities and purity, affecting the material’s natural properties. Utilization of wastewater as feedstock is advantageous as it doesn’t require any pretreatment like lignocellulosic biomass and the process can be more economical. However, long-term sustainability and feasibility must be scrutinized to prevent the further evolution of new pollutants. Further, wastewater characterization and selection of efficient PHA producers with easy and eco-friendly PHA recovery methods are areas that need attention to make the process feasible at a large scale.

## Data Availability

No datasets were generated or analysed during the current study.
